# Macular Pigment Optical Density Is Not Associated with Contrast Sensitivity When Measured with 520 nm Monochromatic Stimuli

**DOI:** 10.3390/vision10040081

**Published:** 2026-10-11

**Authors:** Jeffrey Nightingale, Cameron Wysocky, Jacob Harth, Lisa Renzi-Hammond, Billy R. Hammond

**Affiliations:** 1 Vision Sciences Laboratory, Behavioral and Brain Sciences Program, Department of Psychology, University of Georgia, Athens, GA 30602, USA; jeffrey.nightingale@uga.edu (J.N.); cjw61926@uga.edu (C.W.); 2 Institute of Gerontology, College of Public Health, University of Georgia, Athens, GA 30602, USA; harthjb@uga.edu (J.H.); lrenzi@uga.edu (L.R.-H.)

**Keywords:** contrast sensitivity, macular pigment, spatial vision, dietary carotenoids, retina

## Abstract

Macular pigments (MP; lutein, zeaxanthin, and meso-zeaxanthin) filter short-wavelength light and have been linked to reduced glare and enhanced visual performance. Whether macular pigment optical density (MPOD) is associated with contrast sensitivity (CS) under conditions of minimal MP absorption remains unclear. Fifty-two adults (mean age = 23.5 ± 8.0 years; range = 18–58 years; 85% < 25 years) participated. MPOD was measured using customized heterochromatic flicker photometry. CS was assessed monocularly in the same eye at five spatial frequencies (1.6, 3.2, 8, 16, and 24 cycles/degree), using a custom optical system with narrowband 520 nm laser stimuli. No significant correlations were found between MPOD and CS at any spatial frequency (all |r| ≤ 0.08, all *p* ≥ 0.59), including when individual area estimates were made for each participant’s curve (area under the logged CSF). Separate partial correlations controlling for iris lightness or habitual visual correction were also non-significant. Although MP has been shown to be related to CS when measured under glare or blue haze conditions, these findings suggest that when filtering by MP is minimized, direct effects on CS, especially on the young healthy eye, are likely minimal.

## 1. Introduction

A substantial body of empirical data shows that macular pigments (MPs) influence visual function [1]. The pigments, lutein (L), zeaxanthin (Z), and meso-zeaxanthin (MZ), are concentrated in the inner layers of the macula and screen central photoreceptors (mostly cones) from the bottom third of the visible spectrum (400–520 nm, peak absorbance = 460 nm). The advantage of this selective filtering is particularly evident in bright light [1] and ecologically valid conditions. For example, sunlight is a common source of glare [2] and contains a significant amount of short-wave energy. By filtering such light, MP has been linked to decreasing glare disability/discomfort and accelerating photostress recovery [3,4]. Filtering can also enhance chromatic contrast when short-wave filters selectively reduce blue backgrounds (e.g., blue skylight, which peaks at 460 nm, the peak absorbance of MP) relative to visual targets (mid-to-long wave) [4,5]. Such conditions occur frequently in the natural environment [6].

One of the earliest ideas for how MP improves visual function is called the Acuity Hypothesis [7,8]. The hypothesis was based on the idea that spatial vision (acuity) for polychromatic targets would be improved through limiting the effects of longitudinal chromatic aberration. Although some models were consistent with the hypothesis [9,10], others were not [11,12]. Empirical data [13] suggest that a direct optical effect of MP on contrast sensitivity is minimal. Although filtering by MP could improve spatial vision by reducing factors (e.g., glare or blue haze in the environment) [14] that interfere with the detection of a target, no other optical mechanisms have been proposed that would explain how MP could improve spatial vision directly, especially for achromatic stimuli.

Macular carotenoid supplementation has also been proposed to improve CS through physiological mechanisms, including altered retinal lateral inhibition [15,16]. This interpretation, however, is also not definitive. Parallel changes in CS and the Hermann grid do not establish lateral inhibition as the causal mechanism; both measures, for example, may reflect other neural processes.

Supplementation studies have reported improved CS in some [16,17,18,19,20,21], but not all [22], trials. Longitudinal observational findings in older women have also linked supplement use with greater MPOD and CS [17]. Proposed physiological explanations include effects on retinal health and ocular blood flow [23], but the mechanisms remain uncertain.

In this study, we assessed whether between-person variation in MPOD is correlated with CS using narrowband 520 nm stimuli, for which MP absorption is minimal. A custom-built optical system controlled stimulus wavelength, mean luminance, grating modulation, viewing geometry, and stray light. A previous report using this apparatus found a between-session test–retest reliability of r = 0.85 [24]. In the present study, we did not include a broadband, haze, or glare comparison condition, as these have previously been demonstrated to co-vary with MP (e.g., Stringham et al., [4]), and do not represent isolated CS per se.

## 2. Methods

The target population was adults reporting good ocular health with normal or corrected vision. Fifty-two participants were recruited from students and staff at the University of Georgia (mean age = 23.5 ± 8.0 years; range = 18–58 years; 36 [69%] female, 15 male, and 1 non-binary). Approximately 85% were aged 18–25 years, and four were older than 40. The majority (82%) identified as White; other participants identified as Asian (6%), White-Hispanic (6%), or Black (6%). Visual acuity was initially assessed by self-report and then screened in both eyes using a wall-mounted Snellen chart at 20 feet with habitual correction when worn. All participants met the inclusion criterion of 20/40 or better in both eyes. The majority (58%) did not wear corrective lenses, and 42% used visual correction. Participants who ordinarily wore correction used their habitual correction during CS testing. No objective refraction or assessment of optimal correction at the CS test distance was performed. Iris color was classified using the scheme of Mackey [25]; iris lightness was considered in the covariate analysis. Ocular health was assessed by participant report and acuity screening; participants did not undergo a comprehensive ophthalmologic examination, fundus examination, or OCT. All study procedures and materials were approved by the University of Georgia Institutional Review Board before the study began. All participants gave written and verbal informed consent, and the study adhered to the tenets of the Declaration of Helsinki.

### 2.1. Measurement of Macular Pigment

MP optical density (MPOD) was measured in the eye with better visual acuity (dominant eye if acuity was equal) using customized heterochromatic flicker photometry (cHFP). The apparatus/procedure is detailed in Wooten et al. (1999) [26], with details on the customization process in Stringham et al. (2008) [27]. The background was 6°, 2.75 cd/m^2^, 470 nm, and circular (with a fixation point to the left that was used for the peripheral reference measurement at 7°). The one-degree circular test stimulus consisted of a 460 nm measuring field (peak MP absorbance) alternating with a 570 nm reference field (no MP absorbance). These two fields were superposed and electronically yoked for constant luminance. The light for the system was produced by 20 nm half-bandpass LEDs (Nichia Corp., Mountville, PA, USA) controlled by constant-current, high-frequency electronic pulses. The alternation rate of the measuring and reference fields for both the central foveal and parafoveal reference measurements was carefully optimized for each subject to create a narrow nulling zone. Five trials were collected in each retinal location (central foveal and parafoveal reference at 7° eccentricity), and the participant’s MPOD was calculated based on the difference between the averages of these trials.

### 2.2. Measurement of Contrast Sensitivity

Details of the apparatus and procedure can be found in Hammond et al. (2023) [24]. The CS device was built around two integrating spheres that were constructed from aluminum hemispheres and painted with non-luminescent and highly reflective (98%) white paint (Labsphere, North Sutton, NH, USA). Each hemisphere had input and exit ports (three-inch diameter). The light source was two highly stable 520 nm lasers (Model DS20X90-520-120L; Apinex, Montreal, QC, Canada; apinex.com).

The mean luminance (measured using a United Detector Technology (UDT) S370 Optometer with a photometric lens; Artisan Technology Champaign, IL, USA; artisantg.com) across beams was set at 65 cd/m^2^. Combining light (made Lambertian by internal reflection within the spheres) from the two spheres, in conjunction with a circular neutral density wedge, allowed for continuous variation in the modulation of the sine waves. The UDT S370 Optometer was used throughout the study to monitor the luminance output (half of the combined beam going to the detector and half going to the participant’s eye to maintain constant luminance). The gratings (1.6, 3.2, 8, 16, and 24 cpd on 2 × 2 inch glass made circular with an iris diaphragm) were carefully calibrated by the manufacturer (Applied Image, Rochester, NY, USA; appliedimage.com). The modulation accuracy within the system was also checked by moving a tiny pinhole aperture (~0.5 mm) and a photodetector (ILT 950, International Light Technologies, Peabody, MA, USA; internationallight.com) across and very close to the surface of the grating (just the lowest frequencies were checked) while changing the position of the wedges. The subject’s head position (approximately 89 cm [35 inches] from the exit port) was stabilized with a chinrest and forehead assembly. Optical baffling was used throughout.

On the test day, participants arrived at the test site and completed the informed consent process. All participants were tested on all gratings (to yield contrast sensitivity at 1.6, 3.2, 8, 16 and 24 cpd) in a randomized order. An ascending and descending method of limits was used to evaluate participants’ CS for each grating. Each grating was first presented at near maximum contrast (suprathreshold), and the experimenter confirmed that participants could clearly see the grating. For the descending measures, the experimenter reduced the contrast at a constant rate until the participant was no longer able to resolve the grating. For ascending measures, the experimenter first ensured that participants could not resolve the grating (subthreshold), after which the contrast was steadily increased until the participant reported seeing the grating. Four trials (two ascending, two descending) were completed at each spatial frequency, with rest periods between each grating, for all five spatial frequencies. CS was measured monocularly in the same eye used for MPOD measurement. Investigators were not systematically masked to MPOD values, and MPOD was measured before CS for most participants. Within-session and test–retest CS reliability were not estimated in the present sample.

### 2.3. Statistical Analysis

Bivariate Pearson correlations were used to assess the association between MPOD and CS at each spatial frequency. Separate first-order partial correlations evaluated the association after adjustment for iris lightness or use of habitual visual correction. Visual correction status was a proxy for refractive status rather than a measure of refractive error. Two-sided *p*-values and 95% confidence intervals are reported. Confidence intervals were calculated using Fisher’s z approximation; standard errors were 1/√(*n* − 3) for unadjusted correlations and 1/√(*n* − 4) for first-order partial correlations. No a priori sample size calculation was performed.

We also converted contrast sensitivities measured at 1.6–24 cycles per degree to base-10 logarithms. The area under the log contrast sensitivity function (AULCSF) was then calculated using trapezoidal integration, with log_10_ spatial frequency on the X axis and log_10_ contrast sensitivity on the Y axis. Areas between adjacent measurements were summed over the measured range of 1.6–24 cycles per degree; with this transformation, higher AULCSF values indicate greater overall contrast sensitivity.

## 3. Results

**Figure 1:**
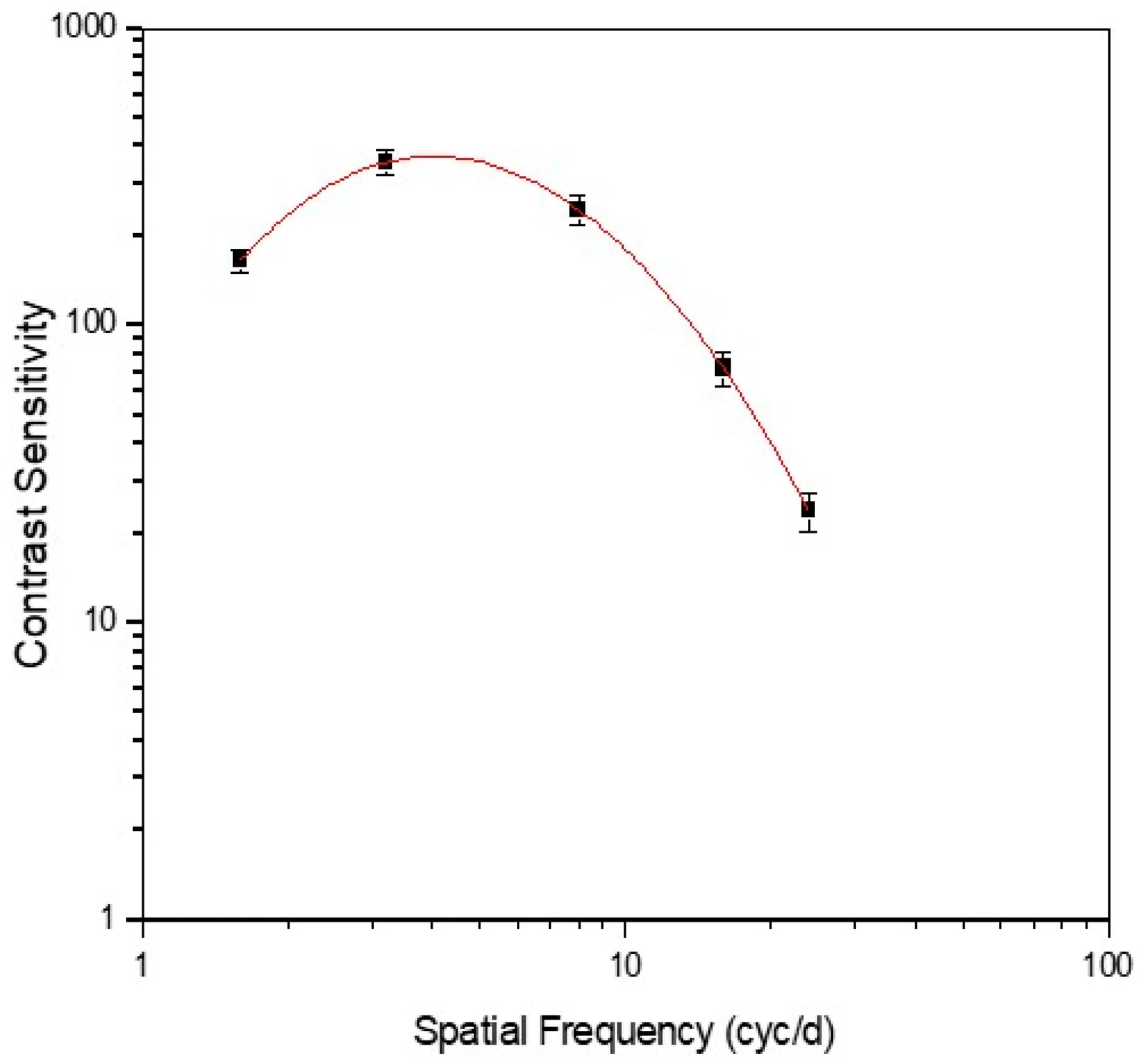
The average and standard error of the mean for the contrast sensitivity values across spatial frequencies.

Table 1 summarizes the participant characteristics available for this report. Table 2 lists the Pearson correlations between MPOD and CS with 95% confidence intervals. Figure 1 shows the mean CS and standard error at each spatial frequency.

Bivariate Pearson correlations between CS and MPOD were non-significant at all five spatial frequencies (Table 2): 1.6 cpd, r(50) = 0.03, *p* = 0.83, 95% CI [−0.24, 0.30]; 3.2 cpd, r(50) = −0.0002, *p* = 0.99, 95% CI [−0.27, 0.27]; 8 cpd, r(50) = −0.08, *p* = 0.59, 95% CI [−0.34, 0.20]; 16 cpd, r(50) = 0.007, *p* 0.96, 95% CI [−0.27, 0.28]; and 24 cpd, r(50) = −0.04, *p* = 0.81, 95% CI [−0.31, 0.24]. With n = 52 and a two-sided α = 0.05, the study had approximately 80% power to detect a correlation of |r| ≈ 0.38. Smaller associations could have been missed, and the confidence intervals do not exclude modest effects.

**Figure 2:**
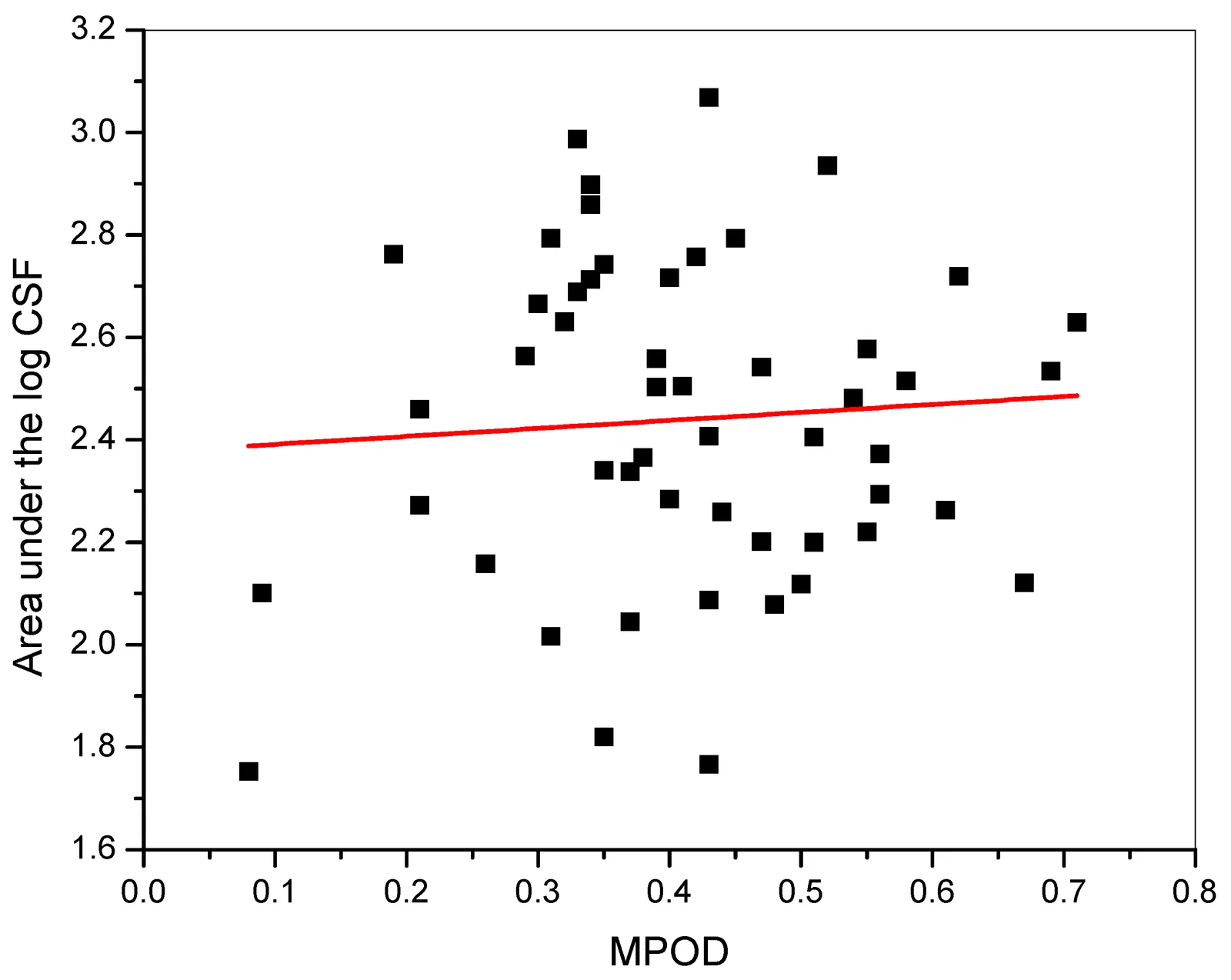
Relationship between macular pigment optical density (MPOD) and area under the log contrast sensitivity function (AULCSF) in 52 subjects. AULCSF was calculated by trapezoidal integration of log_10_ contrast sensitivity against log_10_ spatial frequency over 1.6–24 cycles per degree. Each point represents one subject. MPOD was not significantly associated with AULCSF (Pearson r = 0.068, *p* = 0.630).

We also examined the relationship between MPOD and AUCLF (as shown in Figure 2). MPOD was not significantly associated with overall contrast sensitivity, expressed as AULCSF, in the 52 subjects (Pearson r = 0.068, *p* = 0.630). The fitted linear relationship was nearly flat, with MPOD accounting for less than 1% of the variance in AULCSF.

Separate first-order partial correlations controlling for iris lightness or use of habitual visual correction were also conducted. As shown in Table 3, when controlling for these covariates, the relation between MP and CS remains nonsignificant (although this analysis does not completely correct for confounding by residual refractive error).

## 4. Discussion

MP can enhance chromatic contrast by absorbing more light on one side of a chromatic border than the other [5]. It can also improve achromatic CS in the presence of short-wavelength veiling glare by reducing interfering light [3,4]. For a monochromatic grating, however, uniform attenuation of the luminance maximum and minimum would leave Michelson contrast unchanged. Further, if MP simply reduced mean luminance, such an effect could potentially reduce CS [28]. In this study, we used a narrowband 520 nm stimulus, with an apparatus that carefully controlled mean luminance and stimulus modulation. Our intention was to determine if MP was related to CS when many of the optical conditions that might have driven relations in the past (e.g., filtering of glare, haze, characteristics of the stimuli) were removed.

As shown in the Tables and Figure 2, we did not see an association between between-person differences in MPOD and CS measured with narrowband 520 nm stimuli. The confidence intervals, AULCSF results, and sensitivity analysis, however, indicate that modest associations remain possible. Further, these cross-sectional results cannot be used to infer mechanisms underlying CS changes during supplementation. It does, however, show that MPOD differences maintained over time do not enhance neural contrast sensitivity under conditions which do not favor MP absorption. Time-dependent responses to supplementation could differ from associations with stable tissue levels, but neither those responses nor physiological mechanisms that have been proposed in the past (e.g., effects on lateral inhibition) were tested here. We also did not test retinal protective effects involving oxidative stress and inflammation, which have been proposed in other contexts [29] and may have significant direct effects on CS, especially in older eyes.

Both CS and MPOD have each been used as indicators of visual health [30], but their relationship may depend on the population, stimulus, and testing conditions. Some supplementation trials report CS improvements [16,18,19,20,21], whereas others do not [22]. Stringham et al. [16] reported no association between baseline MPOD and CS, but an association with the change in CS following their supplementation regimen. Parallel changes in Hermann grid perception and CS are consistent with, but do not establish, altered lateral inhibition: the task also involves cortical processes, and the measures could reflect other neural changes.

The present result is consistent with work using absorptive external filters [31], although external filtering is not equivalent to macular filtering. Benefits related to glare reduction or chromatic borders remain compatible with a null association measured at 520 nm without an added glare source.

Several limitations should be considered. The sample was primarily young, and hence the findings may not generalize to older or clinical populations. Ocular health was assessed by self-report and acuity screening, without a comprehensive ophthalmologic examination or OCT, so unrecognized retinal or media abnormalities cannot be excluded. The 20/40 acuity criterion and use of habitual correction do not establish optimal refraction at the approximately 89 cm test distance. Residual blur could particularly affect 16 and 24 cpd and reduce sensitivity to an MPOD association. Both cHFP and the experimenter-controlled method of limits depend on observer responses and are susceptible to measurement variability and criterion effects, especially in inexperienced observers. Investigators were not systematically masked to MPOD, and no independent MPOD or CS reliability estimates were obtained in this sample. The high reliability reported for the optical CS method [24], and for the HFP method of measuring MP [26], does not mean that data from this specific dataset are also reliable.

Whether a greater range of MPOD values (our range was 0.08–0.71), and whether MP could influence CS outside the 1-degree region we measured, are also important questions for future study. Finally, this study did not include broadband, haze, or glare comparison conditions. Those optical scenarios were assessed in past studies [1], which generally show that MP does reduce the influence of factors that interfere with CS performance.

In conclusion, MPOD was not significantly associated with CS at any tested spatial frequency under the narrowband 520 nm condition. These findings do not exclude modest associations or establish the mechanisms of supplementation-related changes. These data were previously presented at the 2024 Vision Sciences Society meeting [32]; the present article provides the full report.

## Figures and Tables

**Table 1:** Participant characteristics.

Characteristic	Value
Participants	52
Age, years, mean ± SD	23.5 ± 8.0
Age range, years	18–58
Aged 18–25 years	Approximately 85%
Older than 40 years	4
Sex	36 female; 15 male; 1 non-binary
Race/ethnicity as reported	White 82%; Asian 6%; White-Hispanic 6%; Black 6%
Habitual visual correction	42% used correction; 58% did not
Visual acuity inclusion criterion	20/40 or better in both eyes
Iris-color categories and counts	Light 19%; Medium 54%; Dark 27%
MPOD mean, SD, range and distribution	$\overset{-}{x}$ = 0.41; SD = 0.14; Range = 0.63 (min 0.08, max 0.71)
AULCSF mean and SD	

**Table 2:** Pearson correlations between CS and MPOD.

Spatial Frequency (cpd)	Correlation (Pearson’s R)	Significance (*p*-Value)	95% CI
1.6	0.03	0.83	[−0.24, 0.30]
3.2	−0.0002	0.99	[−0.27, 0.27]
8	−0.08	0.59	[−0.34, 0.20]
16	0.007	0.96	[−0.27, 0.28]
24	−0.04	0.81	[−0.31, 0.24]

**Table 3:** Separate first-order partial correlations between CS and MPOD controlling for iris lightness or habitual visual correction (df = 49).

cpd	r Value (Iris)	*p* Value (Iris)	95% CI (Iris)	r (Correction)	*P* (Correction)	95% CI (Correction)
1.6	0.03	0.85	[−0.25, 0.30]	0.03	0.83	[−0.24, 0.30]
3.2	0.004	0.98	[−0.27, 0.28]	−0.0006	0.99	[−0.28, 0.28]
8	−0.08	0.56	[−0.35, 0.20]	−0.08	0.59	[−0.34, 0.20]
16	0.0005	0.99	[−0.28, 0.28]	0.008	0.96	[−0.27, 0.28]
24	−0.03	0.85	[−0.30, 0.25]	−0.04	0.80	[−0.31, 0.24]

## Data Availability

The raw data supporting the conclusions of this article will be made available by the authors on request.
